# Factors Associated With Manufacturer Drug Coupon Use at US Pharmacies

**DOI:** 10.1001/jamahealthforum.2021.2123

**Published:** 2021-08-13

**Authors:** So-Yeon Kang, Aditi P. Sen, Joseph F. Levy, Jingmiao Long, G. Caleb Alexander, Gerard F. Anderson

**Affiliations:** 1 Department of Health Policy and Management, Johns Hopkins Bloomberg School of Public Health, Baltimore, Maryland; 2 Center for Drug Safety and Effectiveness, Johns Hopkins Bloomberg School of Public Health, Baltimore, Maryland; 3 Department of Epidemiology, Johns Hopkins Bloomberg School of Public Health, Baltimore, Maryland; 4 Division of General Internal Medicine, Johns Hopkins Medicine, Baltimore, Maryland

## Abstract

**Importance:**

Drug companies offer coupons to lower the out-of-pocket costs for prescription drugs, yet little is known about why they do so for some drugs but not for others.

**Objective:**

To examine whether the following factors are associated with manufacturer drug coupon use: (1) patient-cost characteristics (mean per-patient cost per drug, mean patient copay); (2) drug characteristics (generics availability or “later-in-class-entrant” drugs); (3) drug-class characteristics (in-class coupon use among competitors; in-class generic competition; in-class mean cost and copay).

**Design, Setting, and Participants:**

This was a retrospective cohort analysis of anonymized transactional pharmacy claims sourced from retail US pharmacies from October 2017 to September 2019, supplemented with information derived from Medi-Span, Red Book, and FDA.gov. Data were analyzed from September 2020 to February 2021.

**Main Outcomes and Measures:**

The primary outcome was availability of a manufacturer’s coupon. The secondary outcome was the mean proportion of transactions in which a coupon was used for each product.

**Results:**

The sample of 2501 unique brand-name prescription drugs accounted for a total of 8 995 141 claims. Manufacturers offered a coupon for 1267 (50.7%) of these drugs. When the manufacturer offered a coupon, it was used in a mean (SD) 16.3% (20.3%) of the transactions. Within a drug class, higher mean total cost per patient was positively associated with the likelihood of coupon use (odds ratio [OR], 1.03 per 10% increase; 95% CI, 1.01-1.04), but higher mean patient copay was inversely associated (OR, 0.98; 95% CI, 0.97-0.99). For drug characteristics, single-source later-in-class-entrant products were associated with a greater likelihood of coupon use compared with first entrants and multisource brands (OR, 1.44; 95% CI, 1.09-1.89). The intensity of coupon use was associated with later-in-class-entrant products and the class mean per-patient cost (4.16-percentage-point increase; 95% CI, 1.20-7.13; 0.27 per 10% increase; 95% CI, 0.09-0.44). Drugs with a new in-class brand-name competitor had greater mean coupon use compared with drugs without a new competitor (10.2% of claims with a coupon vs 5.9%).

**Conclusions and Relevance:**

In this cohort study of transactional pharmacy claims, higher mean per-patient total cost within a class was significantly associated with the likelihood of coupon use, but not patient out-of-pocket cost. Manufacturers’ coupons were more likely to be used for expensive later-in-class-entrant products facing within-class competition where coupon use was prevalent.

## Key Points

**Question:**

Why do manufacturers choose to offer coupons for some prescription drugs and not for others?

**Findings:**

In this cohort analysis of 2501 unique brand-name prescription drug products, drug companies offered a coupon for approximately half of the drugs; coupons were likely to be used for later-in-class-entrant products with high total costs in settings where direct competitors also offered coupons. Coupon use was not associated with a given product’s mean out-of-pocket cost.

**Meaning:**

Manufacturer-sponsored coupons were more likely to be used for high-cost later-in-class-entrant products facing within-class competition where coupon use is prevalent.

## Teaser

This cohort study examines whether certain patient-cost characteristics, drug characteristics, and drug-class characteristics are associated with manufacturer drug coupon use.

## Introduction

Given the high out-of-pocket costs of many prescription drugs, many patients use copay offsets to reduce their out-of-pocket costs at the point of sale.^1^ While there are different sources of such offsets,^2^ one common source is coupons offered by pharmaceutical manufacturers. Pharmaceutical manufacturers advertise copay coupons on their websites and various public-facing channels and make them available in physician offices and pharmacies.^3^ One study found that manufacturers increased the number of drugs with coupons offered from fewer than 100 brand-name drugs in 2009 to 700 by 2015.^4^ Given that the US Food and Drug Administration (FDA) approved 234 new drugs between 2009 and 2015, the growth in coupon availability is not likely attributable to the increase in the number of brand-name drugs.^5^ Instead, the rise in coupon availability is likely a response to insurers’ and pharmacy benefit managers’ growing efforts to make consumers more sensitive to differences in out-of-pocket costs within a therapeutic drug class, such as through the use of tiered formularies. Availability of a coupon for a specific drug is dependent on a decision made by the manufacturer to offer a coupon, allocate a budget for the coupon, and determine patient eligibility. Patients then can choose to use a coupon based on their availability.

Despite the wide availability of coupons, little is known about why manufacturers choose to offer coupons for specific drugs and not for others. For example, manufacturers may be motivated to reduce patient out-of-pocket spending to improve patient adherence^6^; alternatively, they may choose to offer coupons to improve their market share within a drug class. Despite previous research on coupon use, little is known about which factors are associated with coupon availability for brand-name drugs. Most studies rely on measuring coupon availability from drug coupon advertisement portals, limiting their ability to capture actual coupon offering by manufacturers.^7,8,9^ Others assess individual-level copay reduction and welfare gain associated with coupon use for a specific subset of drugs.^6,10,11,12,13^ In addition, prior work has tended to focus on off-patent drugs with generic alternatives, yet many brand-name drugs face competition from other brand-name drugs with the same mechanism of action or second-or-later-in-class drugs.^14,15,16,17^ Because manufacturers’ motivation to use coupons may vary by the level of competition each drug faces, it is important to understand the market characteristics when manufacturers choose to offer coupons and when they do not.

We conducted a retrospective cohort analysis of a nationally representative database of anonymized transactional pharmacy claims to characterize factors associated with manufacturers’ choice to provide coupons for prescription drugs in the US. We limited our analyses to coupons for brand-name products. In particular, we examined whether use of manufacturer coupons was associated with the following characteristics: (1) high-cost drugs; (2) single-source later-in-class-entrant drugs; (3) drugs with in-class competitors offering coupons; and (4) high patient copay before coupon.

## Methods

Our analysis was exempt from Johns Hopkins Bloomberg School of Public Health institutional review board approval because it did not constitute human participants research. This study followed the Strengthening the Reporting of Observational Studies in Epidemiology (STROBE) reporting guideline for cohort studies.^18^

### Data and Study Sample

The IQVIA Formulary Impact Analyzer (FIA) was the primary data source. The FIA database is an individual-level, transactional claims database sourced from retail pharmacies. From a 5% nationally random sample of 26 774 102 unique individuals using any copay offset between October 2017 and September 2019, we excluded claims for which a federal health plan was recorded as the payer and identified brand-name prescription drugs with at least 1 transaction where a manufacturer’s product-specific coupon was used. We examined the method of payment and payer names to identify claims using manufacturer drug coupons as either the primary or secondary payer for the transaction.^2^

The claims data were supplemented with information from other sources. Data from Wolters Kluwer Medi-Span provided the wholesale acquisition cost (WAC) of each product at the National Drug Code (NDC) level. The IBM Micromedex Red Book and the FDA’s Orange Book were used to derive drug characteristics, including the availability of generic alternatives, the year of FDA approval, and whether the drug’s primary indication was for an acute or chronic condition.^19^

To determine drug class information, IQVIA’s Uniform System of Classification (USC) was used.^20^ The USC 4 level carries the chemical structure, indication, or method of action of the drug class (eg, dipeptidyl peptidase 4 inhibitor). The USC 4 level was used to identify drugs that share mechanisms of action with other drugs and define the characteristics of the market where the drug resides.

The main data were supplemented with estimated net prices from SSR Health Net Pricing Data, which can be used to estimate manufacturers’ rebates. These data provide estimates of US product level earnings for individual drugs net of all discounts and concessions (eg, drug coupons, rebates, pharmacy discounts) as well as the 340B Drug Pricing Program and rebates given statutorily to state Medicaid agencies. Net prices were available for a subset of brand-name drugs sold by publicly traded companies.^21^

### Outcomes

The focus of the study is which drugs have manufacturer coupons available and, when coupons are offered, what the characteristics of the drugs are. The primary outcome was the presence of any coupon use for each brand-name drug in our sample. The secondary outcome was the intensity of coupon use as measured by the proportion of total product transactions with a coupon for each brand-name drug for drugs with coupon use. Although the secondary outcome is a continuous variable, for descriptive purposes, we categorized drugs with coupons into 4 groups by the quartile of the frequency of coupon use: nearly none (<1.35%), little (<6.5%), moderate (<26.3%), and high (≥26.3%). Drugs without any coupon use were grouped as a fifth category.

### Explanatory Variables

Explanatory variables included patient-cost characteristics, drug characteristics, and market-related characteristics. Patient-cost characteristics included the drug’s mean per-patient total cost (insurer and patient) per drug over the data period and the mean patient copay per claim before coupon. To calculate the drug’s mean per-patient total cost (insurer and patient) per drug, the total units dispensed for each NDC during the study period was multiplied by the WAC, total spending was aggregated across NDC to the level of the product name, and then the total spending on the drug product was divided by the total number of patients who used the product.

Because the net price information was available for only 30% of the study sample, we used net-price-based cost variables for sensitivity analyses. Net-price-based mean per-patient total cost and net-to-gross (WAC) ratios were calculated using the net-price estimates.

Based on previous research suggesting that manufacturers offer coupons in response to the increased competition among substitutable products, we tested the association between coupon availability and drug and drug-class characteristics.^9,13^ Our primary drug characteristics of interest was whether the drug was a single-source later-in-class-entrant, based on generic availability and years since FDA approval by ranking the sequence of drug approvals within the class. Drug-class level characteristics included 3 measures: (1) the drug-class mean proportion of claims with coupons out of total claims dispensed among within-class competitors; (2) the presence of generic competition within the class; and (3) the drug-class mean total cost per patient and patient copay across drugs.

To understand the level of competition among drugs sharing mechanisms of action, we grouped drugs with USC level 4 information into 3 competition categories using the Herfindahl-Hirschman Index (HHI).^20,22^ The HHI was calculated by summing the squares of the market share of an individual product’s total spending at the USC 4 level, taking the total spending in the USC 4 market as the denominator. Drugs were categorized into 4 mutually exclusive groups: monopoly (HHI = 10 000), near monopoly (HHI ≥ 8000), oligopolistic competition (HHI ≥ 2500 and < 8000), and competitive market (HHI < 2500).^23^

In addition, other variables (ie, manufacturer size, market size, and whether the drug’s primary indication is for a chronic condition) that may be associated with coupon use and competition^23^ but not controllable by policy interventions were included as control variables. Manufacturers were categorized into 3 groups: (1) small (<50th revenue percentile); (2) mid-size (<90th percentile); and (3) large (highest 10%). Market size was defined as the total spending in the drug class at the USC 3 level (eg, noninsulin diabetes therapy). We grouped the drugs into small (to 33rd percentile), medium (from 34th to 66th), and large markets (>66th) using tertiles of the market size.

### Statistical Analysis

The unit of analysis was the drug product at the product-name level. Differences between products with and without coupons were identified, and then the cost, product, and drug-class characteristics associated with intensity of coupon use were assessed.

Because the data include variables from different levels (drug-product level and drug-class level), there can be interactions of variables across the levels. To account for variation across drug-class clusters in the likelihood of coupon use and to decompose the within-class and between-class fixed effect, multilevel mixed-effect analyses were used. For the primary outcome of interest, multilevel mixed-effect logistic regressions were fitted to characterize the likelihood of any coupon use. For the secondary outcome of interest, a conditional multilevel mixed-effect generalized linear model with a log-link function and gamma distribution was fitted because the dependent variable—the nonzero proportion of transactions with a coupon among drugs with any coupon use—was positive and right-skewed. Goodness of fit of the models was measured using Akaike Information Criterion and log-likelihood values. The variance inflation factor was calculated for each explanatory variable to examine multicollinearity among variables. Log-transformed cost and copay variables were used because the data were right-skewed.

To decompose the drug’s cost into within-drug-class fixed effects and between-class fixed effects, we estimated the drug-class mean and centered the drug-specific log-transformed cost and copay variables around the mean by subtracting the drug-class mean. In regression analyses, the centered variables were used to estimate whether the drug’s relative cost/copay in a given drug class was associated with the outcomes of interest (within-group effect), and the drug-class mean variables were used to examine whether the drug-class mean between drug-classes was associated with the outcomes of interest (between-group effect).

Clustered SEs at the drug-class level were used to account for the correlation among drugs within the same class. Odds ratios (ORs) were computed to estimate the likelihood of coupon use, and average marginal effects of explanatory variables were computed to estimate the association with coupon use intensity. These can be interpreted as the mean percentage point change in the manufacturer’s coupon use for a change in explanatory variables.

The association of coupon use with various drug characteristics may be affected by a new entrant. Therefore, we estimated whether the recent launch of a new brand-name drug with the same mechanism of action was associated with the change in manufacturers’ coupon use, focusing on nonmonopoly markets. Drugs were stratified into 2 subgroups: one group with a new brand-name drug entering the market after 2015 (n = 1371) and another without a new competitor (n = 1130). Analyses were performed for the full sample as well as both subgroups. Results were considered statistically significant at 2-tailed unpaired *P* < .05. Regression analyses were performed for the full sample and then repeated for drugs with monopolistic competition (monopoly and near monopoly) and high competition (oligopoly and competitive market) to understand the association with the varying level of competition. Analyses were conducted using Stata, version 16.1 (StataCorp LLC).

### Sensitivity Analyses

To assess whether the results from the main model are sensitive to the postrebate cost estimates, regression analyses were repeated with net-price-based cost variable. To test whether the estimates are sensitive to how we modeled the cost and copay measures, models were fitted with or without cost and/or copay variables. Also, to assess whether some small number of outliers in coupon use drove the estimated association of coupon use with explanatory variables, adjusted quantile regression analyses were performed using the 50th, 75th, and 90th percentiles of coupon use frequency as the dependent variable.

## Results

### Manufacturers’ Coupon Use

The sample of 2501 unique brand-name prescription drugs accounted for a total of 8 995 141 claims in the IQVIA data set. More than half of these brand-name products (1267, or 50.7%) had transactions with a manufacturer coupon. Among the 1267 drugs offering a coupon, the mean (SD) percentage of transactions with a coupon was 16.3% (20.3%) ranging from a mean (SD) of 0.6% (0.4%) among drugs in the lowest quartile of coupon use frequency to a mean (SD) of 46.3% (17.3%) among drugs in the highest quartile of coupon use. Drug classes with high frequency of coupon use are summarized in eTable 7 in the Supplement.

### Unadjusted Association Between Product Characteristics and Coupon Use

Table 1 depicts characteristics of products by coupon use (none vs any) and varying levels of manufacturer coupon use intensity (ie, percentage of product transactions with a coupon). Drugs offering coupons were more likely to be single-source drugs than those without coupons (63.0% vs 50.9%) and originate from therapeutic classes with a higher mean prevalence of coupons among in-class competitors (18.6% vs 15.0%). The mean (SD) total cost (including both insurer and patient responsibility) per patient per drug for products without coupon use was $7649 ($33 901), compared with a mean (SD) of $12 165 ($37 601) with coupon use. Companies whose drugs are more expensive relative to the in-class mean were more likely to offer coupons. For example, the mean (SD) total cost was 5% (20%) higher than the class mean among products in the lowest quartile of coupon use frequency and 27% (6%) higher than the class mean among those in the highest quartile of coupon use frequency.

**Table 1.** Characteristics of Prescription Drugs by Manufacturer Coupon Use Level^a^

Variable	All (n = 2501)	None (n = 1234)	Any (n = 1267)	*t* Test *P* value	Group by manufacturer coupon use frequency			
					1st Quartile (n = 317)	2nd Quartile (n = 317)	3rd Quartile (n = 317)	4th Quartile (n = 316)
Mean percentage of claims with coupon use per drug, %, mean (SD)	8.2 (16.6)	0	16.3 (20.3)	NA	0.6 (0.4)	3.4 (1.5)	14.9 (5.5)	46.3 (17.3)
**Patient-cost characteristics**								
Mean total cost per patient per drug, mean (SD), $	10 002 (35 939)^b^	7649 (33 901)^b^	12 165 (37 601)^b^	.003^b^	3593 (11 449)	8115 (20 897)	15 892 (43 077)	21 533 (56 142)
Mean patient copay per claim before offset, mean (SD), $	222 (431)^c^	247 (542)^c^	205 (331)^c^	.03^c^	142 (237)	268 (1305)	249 (376)	267 (547)
0 to ≤100, %	51.1	56.3	47.4	NA	59.9	47.9	43.6	37.8
100 to ≤250, %	28.0	22.1	32.3	NA	29.0	31.1	28.7	40.4
250 to ≤500, %	11.0	9.7	12.0	NA	6.9	13.7	16.5	11.1
>500 or more, %	9.8	11.9	8.3	NA	4.1	7.3	11.3	10.8
**Drug characteristics**								
Single-source drugs, No. (%)	1426 (57.0)	628 (50.9)	798 (63.0)	<.001	154 (48.6)	185 (58.4)	227 (71.6)	232 (73.4)
Me-too single-source drugs, No. (%)	994 (39.7)	434 (35.2)	560 (44.2)	<.001	104 (32.8)	133 (42.0)	164 (51.7)	159 (50.3)
Mean years since the FDA approval, mean (SD), y	14.1 (9.1)	17.0 (10.2)	13.4 (8.6)	<.001	16.3 (9.3)	15.0 (9.0)	11.7 (7.5)	11.6 (8.1)
**Drug-class characteristics**								
Mean prevalence of coupons among in-class competitors, mean (SD), %	16.3 (15.3)	15.0 (15.0)	18.6 (15.1)	<.001	16.2(14.0)	15.3 (13.8)	18.3 (15.2)	24.2 (16.1)
Drugs in classes without generic competition, No. (%)	425 (17.0)	185 (15.0)	240 (18.9)	.009	29 (9.2)	74 (23.3)	75 (23.7)	62 (19.6)
Relative total cost per patient per drug to the class, mean, (SD)^b^	1.00 (1.41)	0.86 (1.62)	1.13 (1.16)	<.001	1.05 (1.20)	1.05 (1.01)	1.17 (1.32)	1.27 (1.06)
Relative patient copay to the drug class, mean (SD)^b^	1.00 (1.21)	1.12 (1.69)	0.92 (0.69)	.001	0.80 (0.65)	0.97 (0.81)	0.97 (0.66)	0.91 (0.57)

Abbreviation: NA, not applicable.

^a^ Analysis of data for October 2017 through September 2019 from IQVIA Formulary Impact Analyzer.

^b^ Calculated without drugs that cost more than $600 000 (0 in “any” group, 5 in “none” group) to mitigate the influence of extreme outlying values on the mean. Before exclusion: 12 164 [SD, 37 601, any] vs 11 019 [SD, 61 030, none], *P* = .59.

^c^ Calculated without drugs that had mean copay more than $5000 (3 in “any” group, 7 in “none” group) to mitigate the influence of extreme outlying values on the mean. Before exclusion: 231 [SD, 744, any] vs 411 [SD, 277, none], *P* = .03.

### Adjusted Association Between Product Characteristics and Coupon Use

Table 2 shows the estimates of the likelihood of coupon use from multilevel mixed-effect logistic regression. Within a drug class, mean total cost per patient was associated with increased coupon availability (OR, 1.03 per 10% increase; 95% CI, 1.01-1.04; *P* < .001). However, mean patient out-of-pocket cost before coupon use was inversely associated with coupon availability (OR, 0.98; 95% CI, 0.97-0.99; *P* = .002).

**Table 2.** Adjusted Likelihood of Manufacturers’ Coupon Use^a^

Variable	Odds ratio (95% CI)	*P* value
Patient-cost characteristics		
Mean total cost per patient per drug (per 10% increase)	1.03 (1.01-1.04)	<.001
Mean patient copay before offset (per 10% increase)	0.98 (0.97-0.99)	.002
Drug characteristics: later-in-class-entrant single-source drugs	1.44 (1.09-1.89)	.01
Drug-class characteristics		
Mean prevalence of coupon use among in-class competitors (per 5-percentage-point increase)	1.18 (1.10-1.26)	<.001
Drug classes without generic competition	1.35 (0.87-2.09)	.18
In-class mean total cost per patient per drug (per 10% increase)	1.01 (1.00-1.02)	.07
In-class mean patient copay before offset (per 10% increase)	0.97 (0.94-1.00)	.06

^a^ Analysis of data for October 2017 through September 2019 from IQVIA Formulary Impact Analyzer.

Within a drug class, later-in-class-entrants were associated with increased coupon availability compared with first-in-class drugs (OR, 1.44; 95% CI, 1.09-1.89). In terms of drug-class characteristics, a drug’s coupon use was associated with in-class competitors’ coupon use. After adjustment, each 5-percentage-point increase in the proportion of transactions with a coupon among in-class competitors was associated with an increased coupon availability (OR, 1.05; 95% CI, 1.09-1.89; *P* = .01).

The likelihood of coupon availability was higher when the in-class mean total cost per patient per drug increased. However, the in-class mean total cost per patient per drug and patient copay were not significantly associated with coupon availability (OR, 1.01; 95% CI, 1.00-1.02; *P* = .07; and OR, 0.97; 95% CI, 0.94-1.00; *P* = .06, respectively).

Among drugs in monopolistic markets, mean total cost per patient per drug was associated with the magnitude of coupon use (OR, 1.06 per 10% increase; 95% CI, 1.02-1.11; *P* < .001) and inversely associated with mean copay (OR, 0.92 per 10% increase; 95% CI 0.87-0.97; *P* = .01) (eTable 1 in the Supplement).

Table 3 shows the estimates of coupon use intensity from the conditional generalized linear model panel for drugs with coupon use. Within a drug class, higher mean total cost per patient than in-class substitutes was associated with higher coupon frequency (mean marginal effect, 0.27-percentage-point increase per 10% increase; 95% CI, 0.09-0.44; *P* = .003). Similarly, later-in-class-entrant single-source drugs were associated with increased coupon use frequency compared with first-in-class or multisource drugs with nonzero coupon use (4.16-percentage-point increase; 95% CI, 1.20-7.13; *P* = .01).

**Table 3.** Mean Percentage Point Change in the Proportion of Transactions With a Coupon Among Drugs With Any Coupon Use^a^

Variable	Change in percentage point of transactions with a coupon (95% CI)	*P* value
Patient-cost characteristics		
Mean total cost per patient per drug (per 10% increase)	0.27 (0.09 to 0.44)	.003
Mean patient copay before offset (per 10% increase)	−0.10 (−0.33 to 0.14)	.42
Drug characteristics: later-in-class-entrant drugs	4.16 (1.20 to 7.13)	.006
Drug-class characteristics		
Mean prevalence of coupon use among in-class competitors (per 5-percentage-point increase)	3.23 (2.38 to 4.08)	<.001
Drug classes without generic competition	3.06 (−1.05 to 7.19)	.15
In-class mean total cost per patient per drug (per 10% increase)	0.23 (0.11 to 0.35)	<.001
In-class mean patient copay before offset (per 10% increase)	−0.65 (−0.27 to 0.14)	.54

^a^ Analysis of data for October 2017 through September 2019 from IQVIA Formulary Impact Analyzer.

### Adjusted Associations Stratifying by Presence or Absence of New Brand-Name Competitor

Drugs with a new in-class brand-name competitor (n = 1371) had significantly greater frequency of coupon use compared with drugs without a new in-class brand-name competitor (n = 1130) (10.2% vs 5.9%; *P* < .001) (Table 4). The likelihood and frequency of coupon use were especially strong among the group of drugs with a new-brand name competitor sharing a mechanism of action. For example, in the stratified multilevel mixed-effect logistic regression, when there was a new brand-name competitor, later-in-class-entrants within a class were associated with greater coupon availability compared with counterparts (OR, 2.63; 95% CI, 1.65-4.20; *P* < .001), while the association was not significant when there was no new brand-name competitor (*P* = .87). Similarly, among the subgroup of drugs with a new brand-name competitor, lack of in-class generic competition was significantly and positively associated with coupon availability (OR, 7.06; 95% CI, 1.57-31.74; *P* = .01), while it was not a significant predictor among the subgroup of drugs without a new brand-name competitor (eTable 2 in the Supplement).

**Table 4.** Subgroup Analysis Among Drugs With Coupon Use—Drugs With vs Without New In-Class Brand-Name Competitor^a^

Variable (referent)	Drugs with new brand-name competitor		Drugs without new brand-name competitor	
	Changes in percentage of transactions with a coupon (95% CI)	*P* value	Changes in percentage of transactions with a coupon (95% CI)	*P* value
Patient-cost characteristics				
Mean total cost per patient per drug (per 10% increase)	0.68 (0.33 to 0.10)	<.001	0.11 (–0.04 to 0.26)	.16
Mean patient copay before offset (per 10% increase)	–0.12 (–0.58 to 0.35)	.62	–0.05 (–0.26 to 0.16)	.65
Drug characteristics: later-in-class-entrant single-source drugs	5.27 (0.23 to 10.31)	.04	1.94 (–1.07 to 4.94)	.21
Drug-class characteristics				
Mean prevalence of coupon use among in-class competitors (per 5-percentage-point increase)	3.17 (2.51 to 3.83)	<.001	2.95 (1.73 to 4.17)	<.001
Drug classes without generic competition	9.78 (0.74 to 18.83)	.03	0.18 (–3.27 to 3.63)	.92
In-class mean total cost per patient per drug (per 10% increase)	0.22 (–0.01 to 0.46)	.06	0.22 (0.10 to 0.33)	<.001
In-class mean patient copay before offset (per 10% increase)	–0.06 (–0.63 to 0.52)	.84	0.05 (–0.17 to 0.26)	.68
Mean percentage of claims with coupons per drug, %, mean (SD)	10.2 (18.3)	NA	5.9 (13.9)	NA

Abbreviation: NA, not applicable.

^a^ Analysis of data for October 2017 through September 2019 from IQVIA Formulary Impact Analyzer.

### Sensitivity Analysis

Descriptive statistics of drugs with estimated net-price information are presented in eTable 3 in the Supplement. In the sensitivity analyses using net-price-based cost estimates, we found an association between the within-class net-price-based cost variable and likelihood of coupon use, but coefficients for other drug-class level variables were not statistically significant, possibly owing to the small number of drug classes (eTable 4 in the Supplement). Using different combinations of cost copay-related variables and specifications and quantile median regressions, we found that our estimates for explanatory variables were consistent across models (eTables 5 and 6 in the Supplement).

## Discussion

In this retrospective cohort analysis of anonymized transactional pharmacy claims sourced in the US, drugs that were later entrants to the market, those that were more expensive than their in-class competitors, or those facing competitors offering coupons were more likely to have greater coupon use. In contrast, drugs with higher mean patient copay were no more likely to be offered coupons than those with lower copay.

These findings suggest that manufacturers use coupons to promote sales of high-cost later-in-class-entrants and to compete against new entrants sharing the same mechanisms of action. It is known that large pharmaceutical companies are increasingly investing in high-price later-in-class-entrants for new lucrative markets, such as immune checkpoint inhibitors, SGLT2 inhibitors, hepatitis C treatments, and HIV antivirals.^15,16^ Consistent with this strategy, we found that in markets with many later-in-class-entrants, manufacturers were sensitive to competitors’ coupon availability.

Recent policy interventions intended to address the adverse effects of manufacturer drug coupons have not taken into account the competition within classes of brand-name drugs. California and Massachusetts prohibit manufacturers from distributing coupons for multisource brand-name drugs, but do not focus on drugs without generic competitors.^24^ There are conflicting policy recommendations on this issue stemming from the lack of consensus on when manufacturers choose to offer drug coupons. Private insurers started implementing copay accumulators to discourage the use of drug coupons by excluding out-of-pocket costs covered by manufacturer coupons from the annual limit on cost-sharing. The Centers for Medicare & Medicaid Services supports the use of copay accumulators for nonfederal governmental plans, such as insurance exchanges. However, these initiatives are hindered at the state level because approximately 20 states are discussing bills to restrict or ban insurers’ use of accumulator programs, and 5 states have enacted these laws, worrying about hikes in patients’ cost burden.^24^ Our study’s findings suggest that coupons are more likely available for patients to afford prescription drugs with alternatives.

### Limitations

Our study has several limitations. First, this is a cross-sectional analysis, and we did not test the causal effect of the explanatory variables on the manufacturer’s coupon use. Second, we did not examine whether coupon use prompted the greater drug utilization, adherence, insurance premiums, or changes in out-of-pocket spending. Third, we did not examine whether any tradeoffs occur between rebates to insurers and coupon discounts to patients because it is not possible to disentangle rebates given to insurers and coupons given to patients from the available net-price estimates. Fourth, our data do not include copay assistance offered by the independent charity patient assistance programs, which are known to cover high-cost specialty drugs.^25^ However, given that charity assistance programs are not designed to cover specific products, the characteristics and intention of offsets may differ from the manufacturer coupons. Lastly, we did not take into account the variation in the comparative therapeutic and economic benefit of each drug. Drugs sharing a mechanism do not mean that the drugs have the same therapeutic benefit, and drugs with a lower therapeutic value may offer more coupons than those with a higher value. However, there is no established systematic method to compare the therapeutic benefit of each drug across classes in the US context.

## Conclusions

In this retrospective cohort analysis of 2501 unique brand-name prescription drug products, manufacturer-sponsored coupons were more likely to be used for expensive later-in-class-entrant products facing within-class competition where coupon use is prevalent. Drug-level mean patient copay was not associated with coupon availability for a specific drug.

## Notes

Supplement. **eTable 1.** Adjusted Likelihood of Manufacturers’ Coupon Use, Drugs in the Monopolistic Market vs. Market With High Competition **eTable 2.** Subgroup analysis – Drugs With vs. Without New In-Class Brand-Name Competitor **eTable 3.** Estimated Net Total Cost Per Patient and Net-to-Gross Price Ratio of Prescription Drugs by Manufacturer Coupon Use **eTable 4.** Adjusted Likelihood of Coupon Use Among Drugs With Coupon Use From Multilevel Mixed-Effect Logistic Regression With Net-Price-Based Cost Variable **eTable 5.** Adjusted Likelihood of Manufacturers’ Coupon Use for Models With Different Specification of Cost-Related Variables (n=2501) **eTable 6.** Mean Percentage Point Changes in the Median of the Proportion of Transactions With Coupons, Quantile Regression (n=2501) **eTable 7.** Drug Classes With High Frequency of Coupon Use
