# Supplementary material for: Factors Associated With Manufacturer Drug Coupon Use at US Pharmacies
Source: JAMA Health Forum. 2021 Aug 13;2(8):e212123. doi: 10.1001/jamahealthforum.2021.2123 (PMC8796883; doi:10.1001/jamahealthforum.2021.2123)
Supplement: Supplement. — eTable 1. Adjusted Likelihood of Manufacturers’ Coupon Use, Drugs in the Monopolistic Market vs. Market With High Competition eTable 2. Subgroup analysis – Drugs With vs. Without New In-Class Brand-Name Competitor eTable 3. Estimated Net Total Cost Per Patient and Net-to-Gross Price Ratio of Prescription Drugs by Manufacturer Coupon Use eTable 4. Adjusted Likelihood of Coupon Use Among Drugs With Coupon Use From Multilevel Mixed-Effect Logistic Regression With Net-Price-Based Cost Variable eTable 5. Adjusted Likelihood of Manufacturers’ Coupon Use for Models With Different Specification of Cost-Related Variables (n=2501) eTable 6. Mean Percentage Point Changes in the Median of the Proportion of Transactions With Coupons, Quantile Regression (n=2501) eTable 7. Drug Classes With High Frequency of Coupon Use [file jamahealthforum-e212123-s001.pdf]

Supplemental Online Content

Kang S-Y, Sen AP, Levy JF, Long J, Alexander GC, Anderson GF. Factors associated with manufacturer drug coupon use at US pharmacies. *JAMA Health Forum*. 2021;2(8):e212123. doi:10.1001/jamahealthforum.2021.2123

eTable 1. Adjusted likelihood of manufacturers' coupon use, drugs in the monopolistic market vs. market with high competition

eTable 2. Subgroup analysis – drugs with vs. without new in-class brand-name competitor

eTable 3. Estimated net total cost per patient and net-to-gross price ratio of prescription drugs by manufacturer coupon use

eTable 4. Adjusted Likelihood of Coupon Use Among Drugs With Coupon Use From Multilevel Mixed-Effect Logistic Regression With Net-Price-Based Cost Variable

eTable 5. Adjusted likelihood of manufacturers' coupon use for models with different specification of cost-related variables (n=2501)

eTable 6. Mean percentage point changes in the median of the proportion of transactions with coupons, quantile regression (n=2501)

eTable 7. Drug classes with high frequency of coupon use

This supplemental material has been provided by the authors to give readers additional information about their work.

© 2021 Kang SY et al. *JAMA Health Network.*

eTable 1. Adjusted likelihood of manufacturers' coupon use, drugs in the monopolistic market vs. market with high competition

	Drugs in the monopolistic market (n=342)			Drugs in the market with high competition (n=2,159)		
Variables	Odds Ratio	p-value	95% CI	Odds Ratio	p-value	95% CI
Patient-Cost Characteristics						
Mean total cost per patient per drug (per 10% increase)	1.06	0.00	1.02-1.11	1.03	0.000	1.01-1.04
Mean patient copay before offset (per 10% increase)	0.92	0.01	0.87-0.97	0.98	0.003	0.97-0.99
Drug Characteristics						
Later-class-entrant single-source drugs				1.44	0.010	1.09-1.90
Drug-class Characteristics						
Mean prevalence of coupon use among in-class competitors (5 percent point increase)				1.18	0.000	1.10-1.26
Drug classes without generic competition				1.37	0.163	0.88-2.14
In-class mean total cost per patient per drug (per 10% increase)				1.01	0.075	0.99-1.03
In-class mean patient copay before offset (per 10% increase)				0.97	0.054	0.94-1.00

Source: Author's analysis of data for October 2017- September 2019 from IQVIA Formulary Impact Analyzer.

© 2021 Kang SY et al. *JAMA Health Network.*

eTable 2. Subgroup analysis – drugs with vs. without new in-class brand-name competitor

	Drugs with new brand-name competitor			Drugs without new brand-name competitor		
Variables (referent)	Odds Ratio	p-value	95% CI	Odds Ratio	p-value	95% CI
Patient-Cost Characteristics						
Mean total cost per patient per drug [a] (per 100% increase)	1.46	0.002	1.16-1.84	1.24	0.003	1.08-1.43
Mean patient copay before offset [b] (per 100% increase)	0.83	0.095	0.66-1.03	0.79	0.010	0.66-0.95
Drug Characteristics						
Later-class-entrant single-source drugs	2.63	0.000	1.65-4.20	1.03	0.867	0.73-1.46
Drug-class Characteristics						
Mean prevalence of coupon use among in-class competitors (5 percent point increase)	1.13	0.011	1.03-1.23	1.19	0.000	1.10-1.29
Drug classes without generic competition	7.06	0.011	1.57-31.74	1.34	0.238	0.83-2.18
In-class mean total cost per patient per drug (per 100% increase)	1.18	0.169	0.98-1.49	1.08	0.375	0.91-1.27
In-class mean patient copay before offset (per 100% increase)	0.86	0.611	0.48-1.53	0.74	0.088	0.53-1.05
Mean percentage of claims with coupons per drug, % (SD)	10.2% (18.3%)			5.9% (13.9%)		

© 2021 Kang SY et al. *JAMA Health Network.*

Among drugs with estimated net-price information, 559 drugs (or 75%) had coupon use, and drugs with coupons had a lower mean net-to-gross ratios (63.4 vs. 71.1, eTable 3).

eTable 3. Estimated net total cost per patient and net-to-gross price ratio of prescription drugs by manufacturer coupon use

Variables	All (n=749)	None (n=190)	Any (n=559)	t-test *p*-value	Group by Manufacturer Coupon Use Frequency			
					1st quartile (n=106)	2nd quartile (n=134)	3rd quartile (n= 159)	4th quartile (n=160)
Estimated mean total net-cost per patient per drug, mean $ (SD)	16097 (43359)	16193 (43542)	16065.4 (43336)	0.9722	4570 (13253)	10177 (20839)	18181 (49799)	26509 (58444)
Estimated mean net-to-gross price ratio per drug, % (SD)	65.5 (26.2)	71.1 (27.4)	63.4 (25.5)	0.0002	63.2 (25.3)	62.3 (25.7)	65.2 (24.8)	62.6 (26.1)
Relative net price-based total cost per patient per drug to the class mean, (SD)[a]	1.00 (1.39)	0.89 (1.32)	1.04 (1.42)	0.2262	0.75 (0.77)	0.83 (0.59)	1.19 (1.65)	1.24 (1.87)

© 2021 Kang SY et al. *JAMA Health Network.*

In the sensitivity analyses using net-price based cost estimates, we found a stronger association between the within-class net-price based cost variable and likelihood of coupon use but coefficients for other drug-class level variables were not statistically significant possibly due to the small number of drug classes (**eTable 4**).

eTable 4. Adjusted likelihood of coupon use among drugs with coupon use from multilevel mixed-effect logistic regression with net-price-based cost variable

	**Main model**			**Net price**			**Net-to-WAC ratio**		
Variables (referent)	Odds Ratio	p-value	95% CI	Odds Ratio	p-value	95% CI	Odds Ratio	p-value	95% CI
Patient-Cost Characteristics									
Mean total cost per patient per drug [a] (per 10% increase)	1.03	0.000	1.01-1.04	1.05	0.001	1.02-1.09			
Mean patient copay before offset [b] (per 10% increase)	0.98	0.002	0.97-0.99	0.94	0.001	0.91-0.98	0.96	0.005	0.93-0.99
Mean net-price based total cost per patient per drug (per 100% increase)									
Net-to-WAC ratio per drug (per 1% increase)							0.80	0.769	0.17-3.66
Drug Characteristics									
Single-source later-class-entrant	1.44	0.011	1.09-1.89	1.45	0.17	0.85-2.48	1.78	0.03	1.07—2.99
Drug-class Characteristics									
Mean prevalence of coupon use among in-class competitors (5 percent point increase)	1.18	0.000	1.10-1.26	1.12	0.051	1.00-1.25	1.13	0.031	1.01-1.26
Drug classes without generic competition	1.35	0.18	0.87-2.09	1.64	0.20	0.77-3.47	1.41	0.31	0.73-2.74
In-class mean total cost per patient per drug (per 10% increase)	1.01	0.07	1.00-1.02						
In-class mean patient copay before offset (per 10% increase)	0.97	0.06	0.94-1.00	0.98	0.230	0.94-1.02	0.97	0.050	0.94 - 1.00
In-class mean net-price-based total cost per patient per drug (per 1% increase)				0.99	0.956	0.98-1.02			
In-class mean net-to-WAC ratio (per 1% increase)							0.74	0.67	0.19-2.92

Source: Author’s analysis of data for October 2017- September 2019 from IQVIA Formulary Impact Analyzer and SSR Health Net Pricing Data

© 2021 Kang SY et al. *JAMA Health Network.*

Using different combinations of cost copay-related variables and specifications, we found that our estimates for explanatory variables were consistent across models (**eTable5**). For example, the odds ratio for later-class-entrant drugs was 1.42 (p=0.003) in the model without cost variables and 1.44 (p=0.01) in the main model.

eTable 5. Adjusted likelihood of manufacturers' coupon use for models with different specification of cost-related variables (n=2501)

Variables	Model 1	Model 2	Model 3	Main model
Patient-Cost Characteristics				
Mean total cost per patient per drug (per 10% increase)	-	1.03***	-	1.03***
Mean patient copay before offset (per 10% increase)			0.99	0.98**
Drug Characteristics				
Single-source later-class-entrant	1.42**	1.43**	1.56**	1.44**
Drug-class Characteristics				
Mean prevalence of coupon use among in-class competitors (5 percent point increase)	1.15***	1.17***	1.20***	1.18**
Drug classes without generic competition	1.40	1.43	1.43	1.35
In-class mean total cost per patient per drug (per 10% increase)		1.01		1.01
In-class mean patient copay before offset (per 10% increase)			0.98*	0.97

* $p < 0.05$, ** $p < 0.01$, *** $p < 0.001$

*Note*s Model 1: Model without cost variables; Model 2: Model 1 with total cost variable; Model 3: Model 1 with copay variable; Model 4: full model with cost and copay variables

© 2021 Kang SY et al. *JAMA Health Network.*

In quantile median regressions to assess whether the outliers drove the estimated association of coupon use explanatory variables, the association was similar across the distribution of coupon use frequency, but there was a greater magnitude of association among drugs with high coupon use frequencies (e.g., change in percentage point of claims with a coupon: 0.37, 2.33, 4.04 in 50th, 75th, 90th percentile) (**eTable 6**).

eTable 6. Mean percentage point changes in the median of the proportion of transactions with coupons, quantile regression (n=2501)

Variables	50th percentile			75th percentile			90th percentile		
	Change in Percent of Transactions with a Coupon (percentage point)	Standard Error	p-value	Change in Percent of Transactions with a Coupon (percentage point)	Standard Error	p-value	Change in Percent of Transactions with a Coupon (percentage point)	Standard Error	p-value
Patient-Cost Characteristics									
Mean total cost per patient per drug (per 1% increase)	0.37	0.13	0.005	2.33	0.68	0.001	4.04	0.96	0.000
Mean patient copay before offset (per 1% increase)	-0.04	0.13	0.768	-1.04	0.84	0.214	-2.51	1.79	0.162
Drug Characteristics									
Me-too single-source drugs	0.93	0.36	0.009	3.85	1.49	0.010	4.78	2.08	0.021
Drug-class Characteristics									
5 percent point increase in mean prevalence of coupons among in-class competitors	0.17	0.08	0.037	1.55	0.50	0.002	3.30	0.58	0.000
Drugs that were more expensive relative to the in-class mean	1.35	0.55	0.014	3.33	1.98	0.093	4.23	2.33	0.070
Drugs that had higher mean patient copay relative to the in-class mean	0.79	0.32	0.014	3.31	1.59	0.037	7.09	2.71	0.009
Drugs that were more expensive relative to the in-class mean	-0.62	0.30	0.038	-1.23	1.42	0.385	-1.99	2.85	0.487

Source: Author’s analysis of data for October 2017- September 2019 from IQVIA Formulary Impact Analyzer.

NOTES: Quantile regression results using median, 75th percentile, and 90th percentile of the coupon use frequency as dependent variable. Coefficients represent changes in percentage point.

© 2021 Kang SY et al. *JAMA Health Network.*

eTable7. Drug classes with high frequency of coupon use

Therapeutic area	Drug Class	Number of products with high coupon use	Number of manufacturers	Mean proportion of transactions with offsets per drug	Mean years on the market
Anti-infectives	Tetracyclines	5	4	61.9	11.3
Non-HIV Antivirals	Viral hepatitis products	9	4	53.6	6.5
Vascular agents	Angiotensin II antagonists	5	4	51.3	11.9
Diabetes, non-insulin	SGLT-2 Inhibitors	9	3	47.3	5.4
HIV antivirals	HIV- reverse transcriptase inhibitor	5	3	44.4	15.8
HIV antivirals	HIV antiviral combinations	12	6	43.7	6.6
Diabetes, non-insulin	DPP-4 inhibitors	7	3	42.1	7.7

*Drug class with more than 5 products with high frequency of coupon use

© 2021 Kang SY et al. *JAMA Health Network.*
